# Distribution of Aldh1L1-CreER^T2^ Recombination in Astrocytes Versus Neural Stem Cells in the Neurogenic Niches of the Adult Mouse Brain

**DOI:** 10.3389/fnins.2021.713077

**Published:** 2021-09-07

**Authors:** Felix Beyer, Wichard Lüdje, Julian Karpf, Gesine Saher, Ruth Beckervordersandforth

**Affiliations:** ^1^Institute of Biochemistry, Emil-Fischer Center, Friedrich-Alexander University of Erlangen-Nürnberg, Erlangen, Germany; ^2^Department of Neurogenetics, Max Planck Institute of Experimental Medicine, Göttingen, Germany

**Keywords:** Aldh1L1, Aldh1L1-CreER^T2^, astrocytes, neural stem cells, subependymal zone, dentate gyrus, neurogenic niche

## Abstract

In the adult central nervous system, neural stem cells (NSCs) reside in two discrete niches: the subependymal zone (SEZ) of the lateral ventricle and the subgranular zone (SGZ) of the dentate gyrus (DG). Here, NSCs represent a population of highly specialized astrocytes that are able to proliferate and give rise to neuronal and glial progeny. This process, termed adult neurogenesis, is extrinsically regulated by other niche cells such as non-stem cell astrocytes. Studying these non-stem cell niche astrocytes and their role during adult neuro- and gliogenesis has been hampered by the lack of genetic tools to discriminate between transcriptionally similar NSCs and niche astrocytes. Recently, Aldh1L1 has been shown to be a pan-astrocyte marker and that its promoter can be used to specifically target astrocytes using the Cre-loxP system. In this study we explored whether the recently described Aldh1L1-CreER^T2^ mouse line (Winchenbach et al., 2016) can serve to specifically target niche astrocytes without inducing recombination in NSCs in adult neurogenic niches. Using short- and long-term tamoxifen protocols we revealed high recombination efficiency and specificity in non-stem cell astrocytes and little to no recombination in NSCs of the adult DG. However, in the SEZ we observed recombination in ependymal cells, astrocytes, and NSCs, the latter giving rise to neuronal progeny of the rostral migratory stream and olfactory bulb. Thus, we recommend the here described Aldh1L1-CreER^T2^ mouse line for predominantly studying the functions of non-stem cell astrocytes in the DG under physiological and pathological conditions.

## Introduction

Astrocytes are key players of central nervous system (CNS) homeostasis. Throughout life, they fulfill a variety of critical functions including but not limited to regulation of blood flow, recycling of neurotransmitters, immune response, energy metabolism, as well as formation and removal of synapses (Prebil et al., 2011; Chung et al., 2015a; MacVicar and Newman, 2015; Weber and Barros, 2015; Colombo and Farina, 2016; Perez-Catalan et al., 2021). Astrocytes are morphologically, molecularly and also functionally heterogeneous. Depending on their localization within different brain areas, astrocytes display region-specific molecular features (Chai et al., 2017; Boisvert et al., 2018; Buosi et al., 2018; Zeisel et al., 2018). There is accumulating evidence that cues from neighboring neurons as well as their activity can influence the molecular and physiological program of astrocytes (Farmer et al., 2016; Hasel et al., 2017; Batiuk et al., 2020; Bayraktar et al., 2020). While important steps have been taken in deciphering the role of astrocytes especially in synapse formation and -removal (Chung et al., 2015a; Allen and Eroglu, 2017), a detailed understanding of how astrocytes perform their many functions, e.g., how they contribute to local neural circuit operations, is still lacking (Khakh and Sofroniew, 2015; Allen and Lyons, 2018). Studying fundamental astrocyte biology and investigating causative and correlative roles of astrocytes in neural circuit function has become even more relevant given their potential contribution to disorders of the CNS (Chung et al., 2015b). To explore astrocyte function *in vivo*, several mouse lines on the basis of the Cre-loxP-system have been developed to specifically target, monitor and manipulate astrocytes utilizing regulatory regions of *Glial fibrillary acidic protein* (*GFAP*; GFAP-Cre; Garcia et al., 2004); human GFAP-Cre (Brenner et al., 1994), *Glutamate Aspartate transporter 1* (*GLAST*; GlastCreER^T2^; Mori et al., 2006), *S100 calcium-binding protein B* (*S100*β; S100β-Cre; Tanaka et al., 2008), and *Connexin 30* (*Cx30*; Cx30-CreER^T2^; Slezak et al., 2007; Yu et al., 2020). While these generated Cre-lines have been successfully used in many studies, the recombination efficiency and specificity has to be carefully considered for each line, developmental stage and brain region when designing a study. Some markers are not expressed in all astrocytes, while others are also expressed in other glial cells. For example, in the adult cortex GFAP is expressed in white matter astrocytes, but not present in gray matter astrocytes (Walz, 2000). In contrast, the *S100*β promoter is active in both gray and white matter cortical astrocytes but also targets oligodendrocyte precursor cells (Deloulme et al., 2004). During the last decades, the field has made great progress in identifying additional astrocyte markers using RNA sequencing techniques. The *aldehyde dehydrogenase 1 family, member L1* (*Aldh1L1*) has emerged as a highly specific pan-astrocyte marker (Cahoy et al., 2008). It is expressed almost exclusively in astrocytes but not in other neural cells, and two *Aldh1L1* promoter-based Cre mouse lines have been developed to study astrocytes in the CNS (Srinivasan et al., 2016; Winchenbach et al., 2016).

Neural stem cells (NCSs) are highly specialized astrocytes that give rise to neuronal and glial progeny during development and at adult stages (Kempermann et al., 2015; Götz et al., 2016). In the adult brain, NSCs (also termed type-B or radial glia-like cells) reside in two discrete neurogenic niches: the subependymal zone (SEZ) of the lateral ventricle and the subgranular zone (SGZ) of the hippocampal dentate gyrus (DG; Kempermann and Gage, 1999; Ming and Song, 2011; Christie et al., 2013; Bond et al., 2015). Neuroblasts (NBs) generated by NSCs of the adult SEZ migrate along the rostral migratory stream (RMS) and differentiate into inhibitory neurons that integrate in existing neuronal circuitries of the olfactory bulb (OB; Lim and Alvarez-Buylla, 2016). In the adult SGZ, NSCs generate glutamatergic neurons that integrate into local hippocampal circuitries (Ming and Song, 2011; Kempermann et al., 2015). Only neurogenic niches constitute the necessary microenvironment for NSC proliferation and maturation of newborn cells (Seidenfaden et al., 2006). Astrocytes are key components of the niches and evolve as important regulators of neurogenesis by controlling NSC activity, fate choice as well as differentiation and survival of neuronal progeny (Sultan et al., 2015; Casse et al., 2018; Schneider et al., 2019). Due to their astrocytic nature, NSCs share a common transcriptional profile with niche astrocytes, including markers such as GFAP and GLAST (Christie et al., 2013). This overlap in gene expression makes it challenging to genetically target astrocytes of the adult neurogenic niches without simultaneously modulating NSCs and their progeny. To understand the function of non-stem cell astrocytes in the adult neurogenic niches the discrimination between niche astrocytes and NSCs is vital. Single-cell RNA sequencing (scRNA seq) analysis of DG cells of postnatal to adult stages (Hochgerner et al., 2018) revealed that Aldh1L1 is expressed in astrocytes but likely absent in NSCs. This observation indicated the possibility to discriminate between astrocytes and NSCs using the recently developed tamoxifen-inducible Aldh1L1-CreER^T2^ transgenic mouse line (Winchenbach et al., 2016). Here, we describe recombination efficiency and specificity of Aldh1L1-CreER^T2^ transgenic mice in the neurogenic niches of the adult brain. Our analysis revealed that Aldh1L1-CreER^T2^ mice efficiently and predominantly recombined in DG astrocytes, targeting only very few radial glia-like NSCs. In contrast, in the SEZ recombination could be observed in astrocytes, ependymal cells, and NSCs, which gave rise to neuronal progeny migrating along the RMS and subsequently differentiating into neurons of the OB.

## Materials and Methods

### Experimental Model

All experiments were carried out in accordance with the European Communities Council Directive (86/609/EEC). Animal experiments were approved by the Governments of Middle Franconia. Animals were group housed in standard cages with *ad libitum* access to water and food and a 12 h light/dark cycle. For all experiments, male and female mice at postnatal day 56 (P56) were used. Aldh1L1-CreER^T2^ mice (Winchenbach et al., 2016) were crossbred to CAG CAT GFP reporter mice (Nakamura et al., 2006). To activate Cre recombinase, animals were injected intraperitoneally with 50 μg/g body weight tamoxifen every 12 h for five consecutive days. All animals have been described previously and all efforts were made to minimize the number of animals used and their suffering.

### Immunohistochemical Staining of Free-Floating Murine Brain Slices

Mice were killed using CO_2_ and transcardially perfused with 50 ml phosphate-buffered saline (PBS, pH 7.4) followed by 50 ml 4% paraformaldehyde (PFA) at the rate of 20 ml/min. Brains were carefully removed and postfixated in 4% PFA at 4°C for 12 h. Subsequently, brains were transferred to a 30% sucrose solution and 40–50 μm thick slices were cut sagittal or coronal at a sliding microtome (Leica Microsystems, Wetzlar, Germany). Immunohistochemical staining were performed on free-floating sections. Slices were washed three times with PBS and incubated with primary antibodies in PBS containing 0.5% Triton X-100 and 3% normal donkey serum for 72 h at 4°C. Following incubation, the tissue was thoroughly washed with PBS at room temperature and subsequently incubated with secondary antibodies in 3% normal donkey serum containing 0.5% Triton X-100 for 24 h at 4°C. As negative controls staining were performed using secondary antibody only. Nuclei were stained with DAPI (1:1,000), washed two times with PBS and sections were mounted on coverslips with Aqua Polymount (Polysciences, Inc.). For immunostaining against Aldh1L1, sections were subjected to antigen retrieval prior to incubation with primary antibodies. For this, slices were incubated in 1 M Tris/EDTA (pH 9) 5 min at 99°C, a cool down step for 20 min at room temperature and washed two times with MilliQ water followed by one washing step with PBS.

Images were taken using a Zeiss inverted Axio Observer 7 with ApoTome.2 equipped with an Axiocam 503, a Colibri 7 LED light source and 20x objective. Confocal images were taken at the Zeiss LSM 780 with four lasers (405, 488, 559, and 633 nm) and 40x and 63x objective lens.

### Antibodies

For immunohistochemical labeling of cells the following primary antibodies were used in this study: Foxj1 (mouse monoclonal, 1:200, ThermoFisher, 14-9965-82, RRID:AB_1548835), GFAP (mouse monoclonal, 1:500, Merck, G3893; RRID:AB_477010), GFP (chicken polyclonal, 1:2,000, Aves, GFP-1020; RRID:AB_10000240), GFP (goat polyclonal, 1:500, Sicgen, AB0066-200; RRID:AB_2333101), Aldh1L1 (rabbit polyclonal, 1:400, Abcam, ab87117; RRID:AB_10712968), Nestin (chicken polyclonal, 1:500, Aves, NES; RRID:AB_10805379), S100 beta (rabbit monoclonal, 1:500, Abcam, ab52642; RRID:AB_882426), DCX (goat polyclonal, 1:500, Santa Cruz, sc-8066; RRID:AB_2088494), and NeuN (mouse monoclonal, 1:200, Merck, MAB377; RRID:AB_2298772). For visualization of primary antibodies the following secondary antibodies (all 1:400) were used: Biotin-conjugated anti-chicken IgY, and anti-goat IgG (donkey polyclonal, Jackson ImmunoResearch, 703-065-155, RRID:AB_2313596, and 705-065-147, RRID:AB_2340397) for amplification of the GFP reporter in combination with Alexa488-conjugated to Streptavidin (1:400; ThermoFisher, S11223); Cy3-conjugated anti-chicken IgY, anti-goat IgG, and anti-mouse IgG (donkey polyclonal, Jackson ImmunoResearch, 703-166-155, RRID:AB_2340364, 705-165-147, RRID:AB_2307351, and 715-165-151, RRID:AB_2315777), Cy5-conjugated anti-rabbit IgG (donkey polyclonal, Jackson ImmunoResearch, 711-175-152, RRID:AB_2340607), and Alexa Fluor 647-conjugated anti-mouse IgG (donkey polyclonal, ThermoFisher, A-31571, RRID:AB_162542).

### Software

Images were processed using Fiji ImageJ and Adobe Photoshop (CS5). Graphs were generated using GraphPad Prism 5.0 software.

## Results

### Aldh1L1 Is Expressed in Astrocytes of Both Adult Neurogenic Niches

**FIGURE 1:**
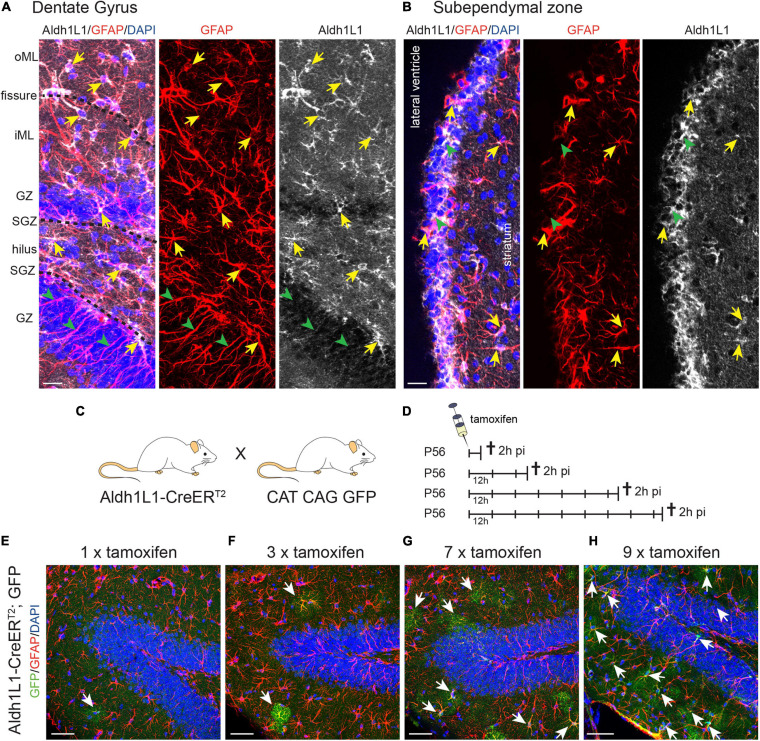
*Aldh1L1 expression in adult neurogenic niches.***(A,B)** Immunohistochemical co-staining of Aldh1L1 and GFAP in the adult mouse neurogenic niches (DAPI in blue, GFAP in red, Aldh1L1 in white). **(A)** Aldh1L1/GFAP double positive astrocytes reside in all DG layers (yellow arrows). Green arrowheads highlight GFAP-positive/Aldh1L1-negative radial processes of adult NSCs in the DG. DG compartment indicated on the left: oML, outer molecular layer; iML, inner molecular layer; GZ, granular zone; SGZ, subgranular zone; scale bar = 20 μm. **(B)** Aldh1L1/GFAP double positive cells residing in the SEZ (yellow arrows). Green arrowheads point toward GFAP-negative/Aldh1L1-positive cells in the SEZ (scale bar = 20 μm). **(C)** Crossing scheme showing the genotype of parental mice used to generate experimental Aldh1L1-CreER^T2^; GFP mice. **(D)** In order to achieve sufficient recombination, we used different tamoxifen pulses in Aldh1L1-CreER^T2^; GFP transgenic mice ranging from one to nine injections every 12 h (12 h) and analyzed recombination 2 h post-injection (2 h pi). **(E–H)** With increasing numbers of tamoxifen injections we observed an increase in the number of recombined astrocytes in the DG of adult Aldh1L1-CreER^T2^; GFP mice (DAPI in blue, GFP in green, GFAP in red; scale bar = 50 μm).

First, we performed immunohistochemical analysis using a specific antibody against Aldh1L1 to determine its expression pattern in both adult neurogenic niches. In the adult DG, we observed that Aldh1L1 is expressed in almost all GFAP^+^ astrocytes of all DG layers (Figure 1A, arrows). Interestingly, radial glia-like NSCs (identified by their GFAP^+^ radial process and a cell body resident in the SGZ) exhibited no or only a very weak Aldh1L1 signal (Figure 1A, arrowheads). This expression pattern fits well to data obtained by scRNA seq of DG cells (Hochgerner et al., 2018), in which the authors identified high mRNA levels of Aldh1L1 in DG astrocytes and only very low levels in NSCs (Supplementary Figure 1A). In the adult SEZ, Aldh1L1 was expressed in most GFAP^+^ astrocytes (Figure 1B, arrows), potentially including radial glia-like NSCs and ependymal cells (Figure 1B, arrowheads). Again these observations are in line with previously published scRNA seq data showing that Aldh1L1 mRNA is present in NSCs and decreases upon further lineage differentiation (Llorens-Bobadilla et al., 2015). A more detailed immunohistochemical analysis will be described in the following chapters.

With the aim to assess whether Aldh1L1 promoter activity can be used to discriminate between astrocytes and NSCs in the adult neurogenic niches, we carried out a comprehensive analysis of the Aldh1L1-CreER^T2^ transgenic mouse line (Winchenbach et al., 2016). To monitor recombination and to fate map NSC progeny, these mice were crossbred to CAG CAT GFP reporter animals (Figure 1C). Intraperitoneal injections of tamoxifen at postnatal day 56 (P56) lead to the excision of a STOP codon and to expression of GFP in cells with an active *Aldh1L1* promoter as well as in their descendants. To establish the minimal effective dose, we injected increasing number of doses of tamoxifen into Aldh1L1-CreER^T2^; GFP mice and assessed the recombination rate 2 h after the last tamoxifen injection (Figure 1D). As exemplified for the DG, one tamoxifen injection resulted in labeling of single astrocytes, which all resided in the inner molecular layer (iML; Figure 1E). Triple tamoxifen injections (every 12 h) more or less doubled the amount of labeled astrocyte, which again were all localized in the iML (Figure 1F). While seven pulses of tamoxifen induced recombination in many more astrocytes, they could still be exclusively found in the iML (Figure 1G) indicating that the *Aldh1L1* promoter exerts the strongest activity in astrocytes of the iML. Only upon nine consecutive tamoxifen injections we observed additional recombination in GFAP^+^ astrocytes of other DG layers, such as granule zone (GZ), SGZ, and hilus (Figure 1H).

### Recombination Efficiency and Specificity in the DG of Adult Aldh1L1-CreER^T2^; GFP Mice

**FIGURE 2:**
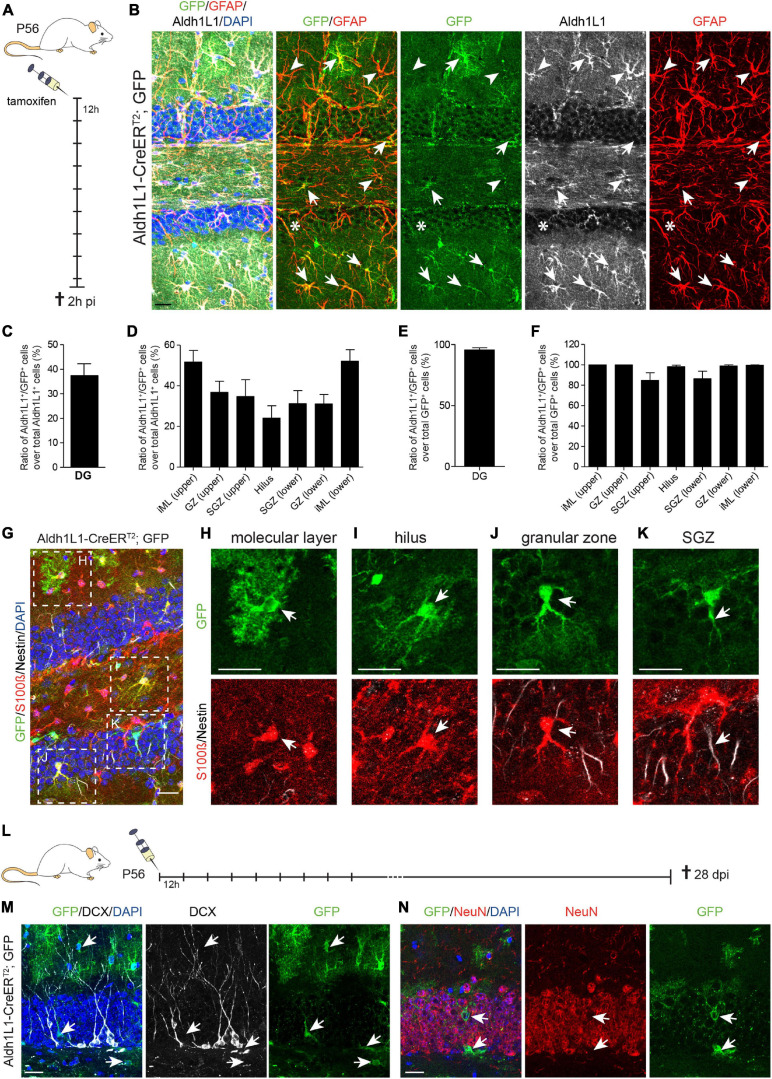
Recombination in Aldh1L1-CreER^T2^; GFP mice efficiently and specifically targets astrocytes but not neural stem cells in the adult dentate gyrus. **(A)** Schematic drawing showing the short-term tamoxifen pulse. Here, Aldh1L1-CreER^T2^; GFP mice were injected with tamoxifen every 12 h (12 h) for five consecutive days and killed 2 h post-injection (2 h pi). **(B)** Representative pictures illustrate an overview of an immunohistochemically stained DG of adult Aldh1L1-CreER^T2^; GFP mice. Arrows point toward GFP/GFAP/Aldh1L1 triple positive cells while arrowheads highlight non-recombined GFAP^+^/Aldh1L1^+^ astrocytes. The asterisk marks a non-recombined GFAP^+^ radial process of an adult NSC (DAPI in blue, GFP in green, GFAP in red, Aldh1L1 in white; scale bar = 20 μm). **(C,D)** The graphs show recombination efficiency in adult DG astrocytes and adult DG astrocyte subtypes (mean + SEM). **(E,F)** The graphs show recombination specificity in adult DG astrocytes and adult DG astrocyte subtypes (mean + SEM). **(G)** Overview image of an immunohistochemically stained Aldh1L1-CreER^T2^, GFP transgenic mouse DG using antibodies against GFP (green), S100β (red), and Nestin (white) 2 h pi; DAPI is depicted in blue, scale bar = 20 μm. **(H–K)** Representative pictures of astrocytes of each DG layer in [**(G)**; boxed areas] were chosen for magnification. Arrows point toward recombined GFP^+^ astrocytes (green) expressing S100β (red) in the molecular layer **(H)**, the hilus **(I)**, the granular zone **(J)**, and the subgranular zone [SGZ; **(K)**] (scale bars = 20 μm). **(L)** Schematic drawing showing the long-term tamoxifen pulse. Here, Aldh1L1-CreER^T2^; GFP mice were injected with tamoxifen every 12 h (12 h) for five consecutive days and killed 28 days post-injection (dpi). **(M,N)** Representative pictures of immunohistochemical staining against GFP (green) and DCX (white) and NeuN (red), respectively. Arrows point toward recombined GFP^+^ cells, which do not co-express either DCX or NeuN; DAPI is depicted in blue; scale bar = 20 μm.

Next, we assessed recombination efficiency and specificity in the adult DG of Aldh1L1-CreER^T2^; GFP reporter mice, which received 9x tamoxifen every 12 h, and were killed 2 h post induction (2 h pi; Figures 2A,B and Table 1). We used the ratio of immunolabeled Aldh1L1^+^ cells that were co-labeled with the GFP reporter over all Aldh1L1^+^ cells as a measure of recombination efficiency. Immunohistochemical analysis on sagittal brain sections revealed that almost all GFP-reporter^+^ cells co-expressed the astrocyte markers GFAP and Aldh1L1 across all DG layers (Figure 2B, arrows; Supplementary Figures 1C–H). However, recombination occurred only in about 40% of Aldh1L1^+^ astrocytes (Figure 2B, arrows, and Figure 2C). This efficiency ranged from 24.1% (hilus) to 52.1% (lower iML; Figure 2D) most likely due to differential activity of the *Aldh1L1* promoter in astrocytes of distinct DG compartments. In accordance with little to no expression of Aldh1L1 protein and mRNA; Supplementary Figure 1A) in NSCs, we observed only very few radial glial-like NSCs expressing the GFP-reporter (Figure 2B, asterisk). Specificity of the genetic labeling was determined as the ratio of Aldh1L1/GFP double positive cells over all GFP^+^ (= recombined) cells. The overall recombination specificity in the DG was close to 100% (95.4%; Figure 2E). Here, recombination occurred to 100% in astrocytes in all DG regions except the SGZ, in which recombination specificity was only ≈85% (Figure 2F). To assess the identity of the GFP^+^ cells that were not labeled by anti-Aldh1L1 antibody, we stained against NSC-marker Nestin together with S100β, which is expressed only in postmitotic astrocytes of the neurogenic niches. Confirming the astrocytic identity of recombined cells, the vast majority of GFP^+^ cell in iML, GZ and hilus expressed S100β (Figures 2G–J). However, the co-expression of GFP and S100β was less in the SGZ (Supplementary Figure 1I), where a small percentage of GFP^+^ cells co-expressed Nestin (<1.5%; Supplementary Figure 1J) and acquired radial glia-like morphology (Figure 2K). Even though the percentage of recombined NSCs (GFP^+^/Nestin^+^) over all NSCs (Nestin^+^) was less than 1.5% (Supplementary Figure 1K), we wanted to determine to which extent they contribute to the generation of adult-born neurons. To address this question, we used a tamoxifen protocol in which Aldh1L1-CreER^T2^; GFP transgenic mice were injected nine times with tamoxifen for five consecutive days and killed 28 days post-injection (dpi; Figure 2L). If the recombined NSCs generated neuronal progeny, we expected to see GFP-reporter^+^ NBs (identified by DCX) or neurons (NeuN^+^) in the DG. However, virtually none of the recombined cells in the DG co-expressed DCX or NeuN 28 dpi (Figures 2M,N, respectively) indicating that the few initially recombined NSCs did not give rise to neuronal progeny. In sum, Aldh1L1-CreER^T2^ mice showed a high recombination efficiency and specificity in niche astrocytes indicating that this mouse line is very well suited to functionally target niche astrocytes in the DG.

**TABLE 1:** Recombination in neurogenic niches of adult Aldh1L1-CreER^T2^ transgenic mice.

**Brain region**	**Time point post tamoxifen injection**	**Marker protein**	**Mean of co-labeled cells % of all GFP^+^ cells)**	**Number of analyzed GFP^+^ cells**
Subependymal zone	2 h pi	Aldh1L1	39.3	618
		Nestin	34.3	300
		S100b	34.9	300
	28 dpi	DCX	43.4	584
		NeuN	0.8	584
Dentate gyrus	2 h pi	Aldh1L1	95.4	2,487
		Nestin	1.4	995
		S100b	70.3	995
	28 dpi	DCX	0.01	2,558
		NeuN	0	2,558
Rostral migratory stream	28 dpi	DCX	46.1	352
		NeuN	0.5	352
Olfactory bulb	28 dpi	DCX	25.7	1,031
		NeuN	36.5	1,031

*Quantitative immunohistochemical analysis of adult Aldh1L1-CreER^T2^; CAG CAT GFP animals. Values in the table represent the average number of double positive (GFP reporter plus cell type specific marker protein) cells over all GFP^+^ cells at a certain time point and in a defined location in the brain. For each time point, location, and marker protein cells were counted on 30–40 μm thick z-stack images and three to four sections for *n* = 3–6 animals.*

### Recombination in the SEZ of Adult Aldh1L1-CreER^T2^ Transgenic Mice

**FIGURE 3:**
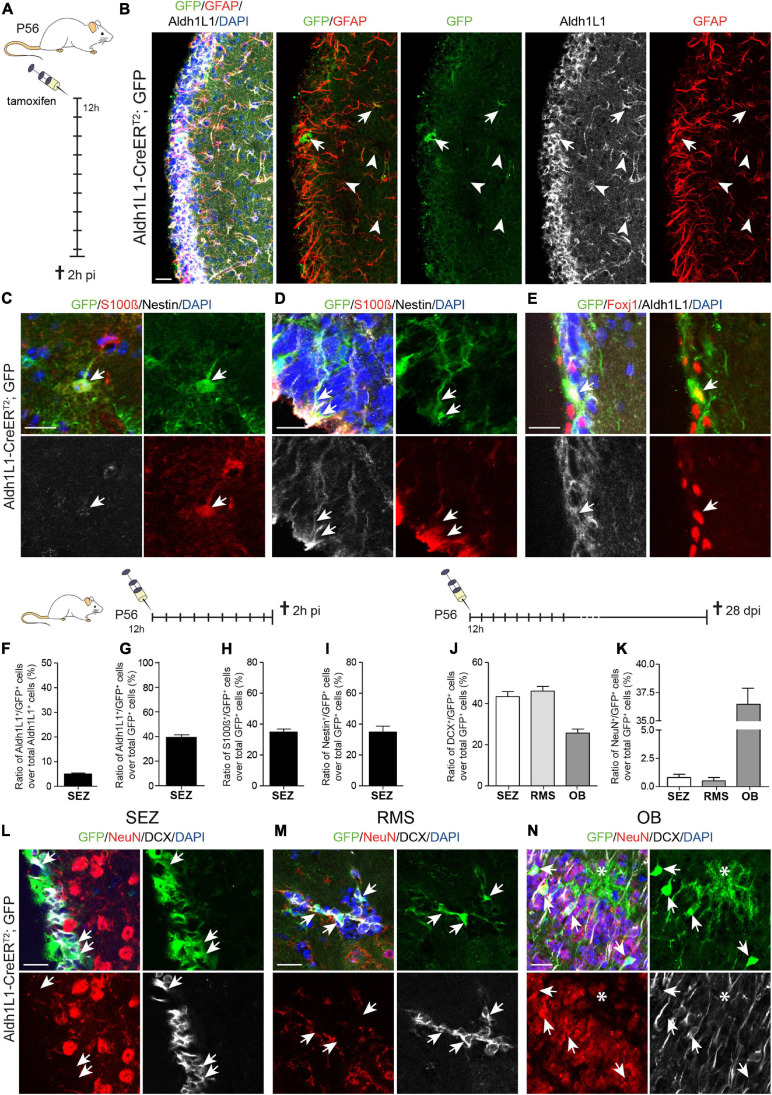
Recombination in Aldh1L1-CreER^T2^; GFP mice targets astrocytes and neural stem cells in the subependymal zone. **(A)** Schematic drawing showing the short-term tamoxifen pulse. Here, Aldh1L1-CreER^T2^; GFP mice were injected with tamoxifen every 12 h (12 h) for five consecutive days and killed 2 h post-injection (2 h pi). **(B)** Representative pictures illustrate an overview of the immunohistochemically stained SEZ of adult Aldh1L1-CreER^T2^; GFP mice. Arrows point toward GFP/GFAP/Aldh1L1 triple positive cells while arrowheads highlight non-recombined GFAP^+^/Aldh1L1^+^ cells (DAPI in blue, GFP in green, GFAP in red, Aldh1L1 in white; scale bar = 20 μm). **(C,D)** Representative images show an immunohistochemically stained SEZ of Aldh1L1-CreER^T2^, GFP transgenic mice using antibodies directed against GFP (green), S100β (red), and Nestin (white). The arrows point toward a GFP/S100β double positive astrocyte in the SEZ **(C)**, or a triple positive radial glia-like NSC **(D)** DAPI is depicted in blue, scale bar = 20 μm. **(E)** Images show an immunohistochemically stained SEZ of Aldh1L1-CreER^T2^, GFP transgenic mice using antibodies directed against GFP (green), FoxJ1 (red), and Aldh1L1 (white). The arrows point toward a triple positive ependymal cell. DAPI is depicted in blue, scale bar = 20 μm. **(F,G)** Efficiency and specificity of recombination are expressed as the ratio of Aldh1L1/GFP double positive cells over all Aldh1L1^+^ or GFP^+^ cells, respectively (mean + SEM; 2 h pi). **(H)** Graph showing the ratio of S100β/GFP double positive cells over all recombined (GFP^+^) cells (mean + SEM; 2 h pi). **(I)** Graph showing the ratio of Nestin/GFP double positive NSCs over all recombined (GFP^+^) cells (mean + SEM; 2 h pi). **(J–N)** Using the long-term tamoxifen protocol (28 dpi) and immunohistochemical analysis with antibodies against GFP (green), DCX (white) and NeuN (red) we assessed the generation of neuronal progeny from recombined NSCs in the SEZ **(L)**, the RMS **(M)** and the OB **(N)**. Data in panels **(J,K)** represents mean + SEM. Arrows point toward GFP^+^ cells co-expressing either DCX **(L,M)** or NeuN **(N)**. Asterisk in panel **(N)** highlights a recombined astrocyte with protoplasmic morphology in the OB (DAPI is depicted in blue, scale bar = 20 μm).

In addition to the analysis in the DG, we assessed recombination efficiency and specificity in the adult SEZ of Aldh1L1-CreER^T2^; GFP mice (Table 1). Likewise, this characterization was performed by immunohistochemistry against the Cre reporter GFP and cell type specific markers GFAP, Aldh1L1, S100β, Nestin, FoxJ1 and DCX in animals killed 2 h after the last tamoxifen injection (Figure 3A and Supplementary Figure 2A). Our first observation was that much less recombination events (GFP^+^ cells) occurred in the SEZ compared to the DG (Figures 3B–F). Even though Aldh1L1 is expressed in basically all GFAP^+^ astrocytes at levels comparable to DG astrocytes, most astrocytes did not recombine, leading to recombination efficiency of only 5% (Figure 3F). Furthermore, the expression of Aldh1L1 was not specific to astrocytes but also detectable in other cells (Figure 3B). Using S100β to identify astrocytes, Nestin to label NSCs and ependymal cells, and FoxJ1 as an ependymal-specific marker, we determined that the recombined cells represented a mixture of astrocytes (Figure 3C, arrow), radial glia-like NSCs (Figure 3D, arrows), and ependymal cells (Figure 3E, arrow). Quantifying the recombination specificity we observed that only 39.3% of the recombined cells expressed Aldh1L1 (Figure 3G), and only 34.9% of reporter-positive cells expressed S100β (Figure 3H) indicating that the majority of recombined cells were not astrocytes. Almost the same number of GFP^+^ cells expressed Nestin (34.4%; Figure 3I), which is expressed in ependymal cells and SEZ NSCs. In line with scRNA seq data (Llorens-Bobadilla et al., 2015), NBs do not express Aldh1L1 (Supplementary Figure 2B) and we found only very few DCX^+^ NBs amongst the recombined cells (Supplementary Figure 2C).

To determine to which extent recombined NSCs contribute to neurogenesis, we analyzed tamoxifen induced Aldh1L1-CreER^T2^; GFP mice at 28 dpi and stained for DCX and NeuN to assess the number of GFP^+^ immature and mature neurons in the SEZ, RMS and OB (Figures 3J–N). In contrast to the DG, 43.4% and 46.1% of recombined cells in the SEZ and RMS co-expressed DCX, respectively, revealing their identity as immature neurons (Figures 3J,L,M and Supplementary Figure 2E). In the OB, 25% of GFP^+^ cells were labeled with DCX (Figures 3J,N and Supplementary Figures 2F,G), while 36.5% acquired already a more mature stage and expressed NeuN (Figures 3K,N and Supplementary Figure 2G). Among all GFP^+^ cells in the OB some cells did neither express DCX nor NeuN and exhibited the protoplasmic morphology of astrocytes (Figure 3N, asterisk; OB overview in Supplementary Figure 2F), which is in line with earlier findings revealing recombination in OB astrocytes using an Aldh1L1-GFP mouse line (Lozzi et al., 2020). Taken together, the efficiency of Aldh1L1-CreER^T2^ mice in the adult SEZ is very low and recombination is not specific to niche astrocytes, but also includes ependymal cells and NSCs giving rise to neuronal progeny. Hence, this mouse line cannot be used to functionally address niche astrocytes in the SEZ.

## Discussion

Work of the past decades identified numerous signaling pathways, transcriptional and epigenetic regulators as well as cellular constituents of the distinct neurogenic niches to exert tight control over the process of adult neurogenesis. While especially radial glia-like NSCs have been extensively investigated, more recent data indicate that also niche astrocytes constitute important regulators of neurogenesis (Schneider et al., 2019). However, we are just at the beginning to understand how astrocytes maintain niche homeostasis and regulate neurogenesis in the adult brain. Considering their potential contribution to neurodegenerative and neuropsychiatric disorders (Chung et al., 2015b), further investigations of non-stem cell astrocytes is crucial to develop new astrocytes-based therapies against such CNS disorders (Acioglu et al., 2021). Using existing Cre mouse lines based on promoter activity of *Glast* or *GFAP* (Brenner et al., 1994; Garcia et al., 2004; Mori et al., 2006) it was so far not possible to discriminate niche astrocytes from adult NSCs due to their highly overlapping transcriptional profile. With the aim to identify a Cre mouse line to monitor and functionally target only non-stem cell astrocytes, we characterized a recently generated BAC-transgenic mouse line, which uses the *Aldh1L1*-promoter to drive tamoxifen-inducible Cre recombinase expression (Winchenbach et al., 2016). Aldh1L1 has been found to be highly expressed in CNS parenchymal astrocytes (Cahoy et al., 2008) and CreER^T2^ reporter analysis recently revealed efficient and specific recombination in astrocytes of the adult cortex, cerebellum, corpus callosum and fimbria (Winchenbach et al., 2016). However, recombination of this mouse line in the adult neurogenic niches has not been investigated yet. Previous analysis of a non-inducible Aldh1L1-Cre line led to the conclusion that recombination also occurs in postnatal NSCs (Foo and Dougherty, 2013) of both niches. Here, recombined neurons were identified in the GZ and RMS, which most likely represent rather the progeny of NSCs, which recombined during developmental stages. In our work, we comprehensively investigated if Aldh1L1 is expressed by adult NSCs in the neurogenic niches, and to which extent Aldh1L1-dependent recombination induced during adulthood targets NSCs of the adult SEZ and SGZ in Aldh1L1-CreER^T2^ mouse line generated by Winchenbach et al. (2016).

In the adult SEZ, we observed recombination in astrocytes and NSCs as well as in ependymal cells. Moreover, recombined NSCs in the SEZ gave rise to neuroblasts, which migrated along the RMS and eventually differentiated into GFP-positive neurons in the OB. This is in line with scRNA seq analysis of early stages of the neurogenic lineage in the SEZ, showing that Aldh1L1 mRNA is expressed in NSC stages and gets downregulated in NBs (Llorens-Bobadilla et al., 2015). In contrast, we found almost no radial glia-like NSCs that underwent recombination in the adult DG. Instead, Aldh1L1-CreER^T2^ transgenic animals predominantly target niche astrocytes in the adult DG. Also for the DG, our results are in accordance with scRNA seq data, which reported high Aldh1L1 mRNA levels in astrocytes in contrast to very low Aldh1L1 levels in NSCs (Hochgerner et al., 2018). Thus, recombination specificity strongly matched the endogenous expression of Aldh1L1. However, it is important to note that our analysis was carried out using only CAG CAT GFP reporter mice to visualize recombination events. Hence, it cannot be ruled out that other, more sensitive reporters, such as tdTomato (Winchenbach et al., 2016), may reveal a higher recombination rate in NSCs, even though DG radial glia-like NSCs only show negligible levels of Aldh1L1 expression. Targeting almost exclusively niche astrocytes in the DG represents an important step forward to label and functionally assess their role in hippocampal circuit formation and operation as well as in adult neurogenesis and hippocampal plasticity. Furthermore, it offers the possibility to genetically modify niche astrocytes in pathological conditions in order to develop new therapeutic strategies to treat CNS disorders.

Overall, the recombination efficiency of Aldh1L1-CreER^T2^ mice in DG astrocytes was <40%. While it is possible to further increase recombination efficiency by using stronger Tamoxifen-induction protocols (Winchenbach et al., 2016), it overall matched those observed in other astrocyte-specific Cre-driver lines, such as Glast:CreER^T2^ and Connexin30-CreER^T2^ (Slezak et al., 2007). All analyzed Cre-driver lines revealed differences in the recombination rate ranging from 20 to 90% in dependence of the brain region analyzed (Slezak et al., 2007). This observation already indicates that astrocytes from different brain areas are heterogeneous. In the last years, our knowledge about astrocyte diversity has been constantly extended. Especially the development of scRNA seq allowed comprehensive insights into molecular diversity of neural cells, including astrocytes (Dulken et al., 2017; Hochgerner et al., 2018; La Manno et al., 2018; Zeisel et al., 2018; Hodge et al., 2019; Batiuk et al., 2020; Bayraktar et al., 2020). These studies provide additional evidence that astrocytes are developmentally specified (Zeisel et al., 2018), and astrocyte subclasses populate distinct brain areas where they exert region-specific molecular function (Chai et al., 2017; Boisvert et al., 2018; Buosi et al., 2018). Furthermore, also within the same region, astrocytic differential mRNA and protein expression can predict specific function to support local circuitries (Batiuk et al., 2020; Bayraktar et al., 2020). Interestingly, we observed that even though recombination specificity was equally high in astrocyte subpopulations of all DG compartments (iML, GZ, SGZ, and hilus), substantial differences in their recombination efficiency could be detected indicating intra-regional astrocyte diversity. This is supported by our own unpublished data in which we observed that astrocytes in the adult DG differ in morphology, molecular profile and functional properties depending on the DG compartment that they reside in. Highlighting the role of distinct astrocyte subtypes of different DG layers, a recent publication showed that adult DG astrocytes contribute to disease conditions in a subtype-specific manner. Here, tau protein accumulates in hilus astrocytes of individuals with Alzheimer’s disease, and overexpression of 3R tau specifically in hilus astrocytes by a lentiviral approach is sufficient to induce AD-like symptoms such as neuronal dysfunction and memory deficits (Richetin et al., 2020). The here described Aldh1L1-CreER^T2^ mouse line represents a valuable tool to better understand the role of DG astrocytes in health and disease. Evolving evidence supports a functional role of astrocytes in the development of pathological states of nervous system disorders (Chung et al., 2015b). With the long-term goal to utilize the astrocytes’ potential for novel therapeutic strategies, it may become important to even target astrocyte subtypes within a given region. Therefore, there is a strong need to further improve the genetic and molecular toolbox to refine more specific strategies to eventually target astrocyte subtypes.

## Data Availability Statement

The raw data supporting the conclusions of this article will be made available by the authors, without undue reservation.

## Ethics Statement

The animal study was reviewed and approved by Governments of Middle Franconia.

## Author Contributions

FB and RB: conceptualization, formal analysis, resources, funding acquisition, and writing—original draft. FB, WL, JK, GS, and RB: investigation. All authors contributed to manuscript revision, read and approved the manuscript.

## Conflict of Interest

The authors declare that the research was conducted in the absence of any commercial or financial relationships that could be construed as a potential conflict of interest.

## Publisher’s Note

All claims expressed in this article are solely those of the authors and do not necessarily represent those of their affiliated organizations, or those of the publisher, the editors and the reviewers. Any product that may be evaluated in this article, or claim that may be made by its manufacturer, is not guaranteed or endorsed by the publisher.
