# Supplementary material for: Distribution of Aldh1L1-CreERT2 Recombination in Astrocytes Versus Neural Stem Cells in the Neurogenic Niches of the Adult Mouse Brain
Source: Front Neurosci. 2021 Sep 7;15:713077. doi: 10.3389/fnins.2021.713077 (PMC8452868; doi:10.3389/fnins.2021.713077)
Supplement: Supplementary file 1 [file Data_Sheet_1.pdf]

Supplemental information

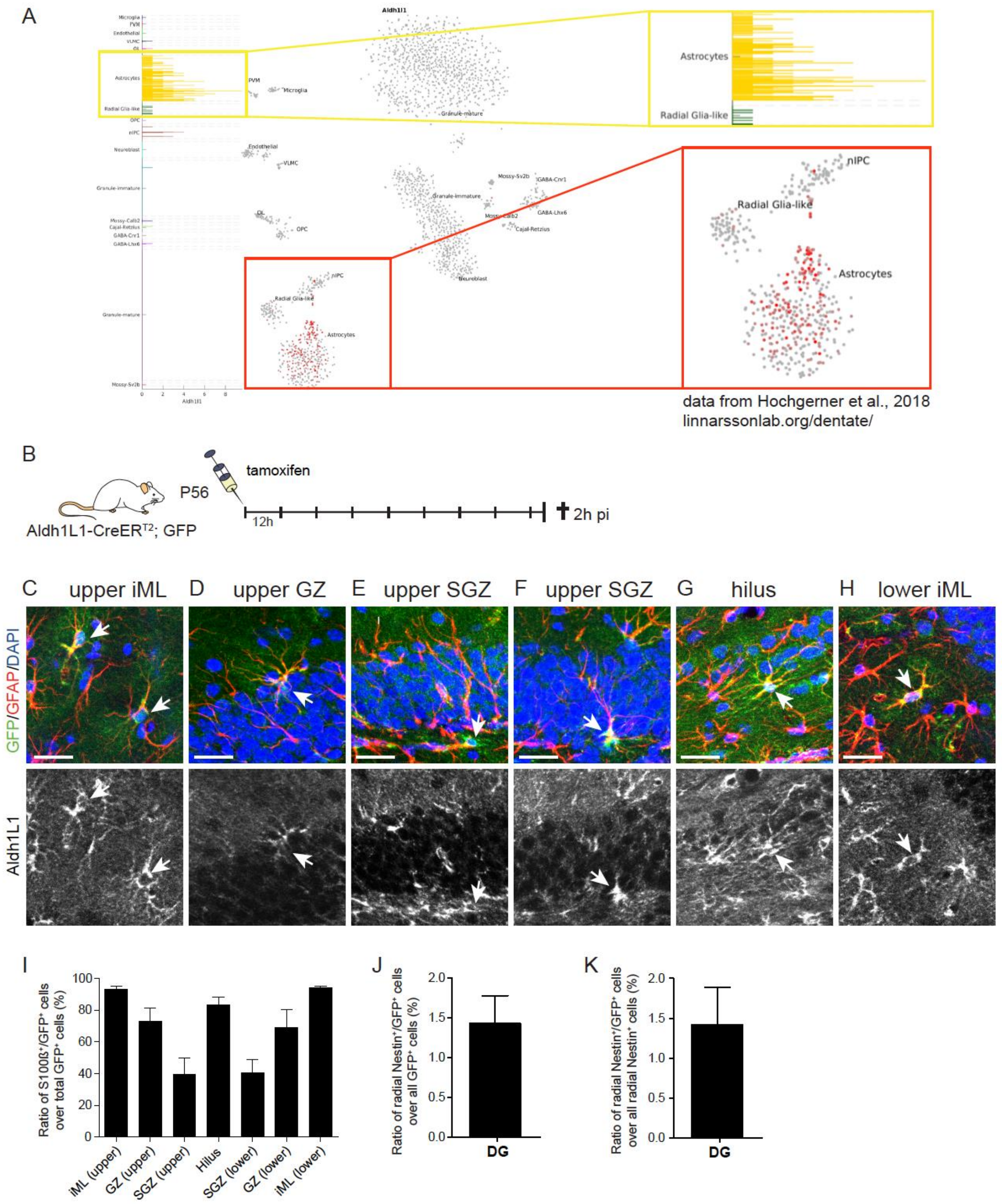

Supplemental figure 1: Recombination of Aldh1L1-CreER^{T2}; GFP mice in the hippocampus.

(A) tSNE-plot from scRNA seq analysis of DG cells (Hochgerner et al.) showing robust *Aldh1L1* mRNA expression in DG astrocytes and only sparse expression in radial glia-like NSCs and neuronal intermediate progenitor cells (nIPC). (B) Schematic drawing showing the short-term tamoxifen pulse. Here, Aldh1L1-CreER^{T2}; GFP mice were injected with tamoxifen every 12

hours (12h) for five consecutive days and killed 2 hours post-injection (2h pi). (C-H) Images showing examples of recombined GFP-reporter^{+} cells in each DG compartment (indicated above). Arrows in representative pictures point towards GFP^{+} astrocytes (green); DAPI = blue, GFAP = red; Aldh1L1 = white (below); all scale bars = 20 µm). Please note a rare recombination event in a radial glia-like adult NSC in (F). (I) Graphs show the ratio of S100β/GFP double positive astrocytes over all recombined (GFP^{+}) cells in all regions of the adult DG (mean + SEM; 2h pi). (J) Graph showing the ratio of Nestin/GFP double positive NSCs with radial morphology over all recombined (GFP^{+}) cells (mean + SEM; 2h pi). (K) Graph showing the ratio of Nestin/GFP double positive NSCs with radial morphology over all radial Nestin^{+} NSCs (mean + SEM; 2h pi).

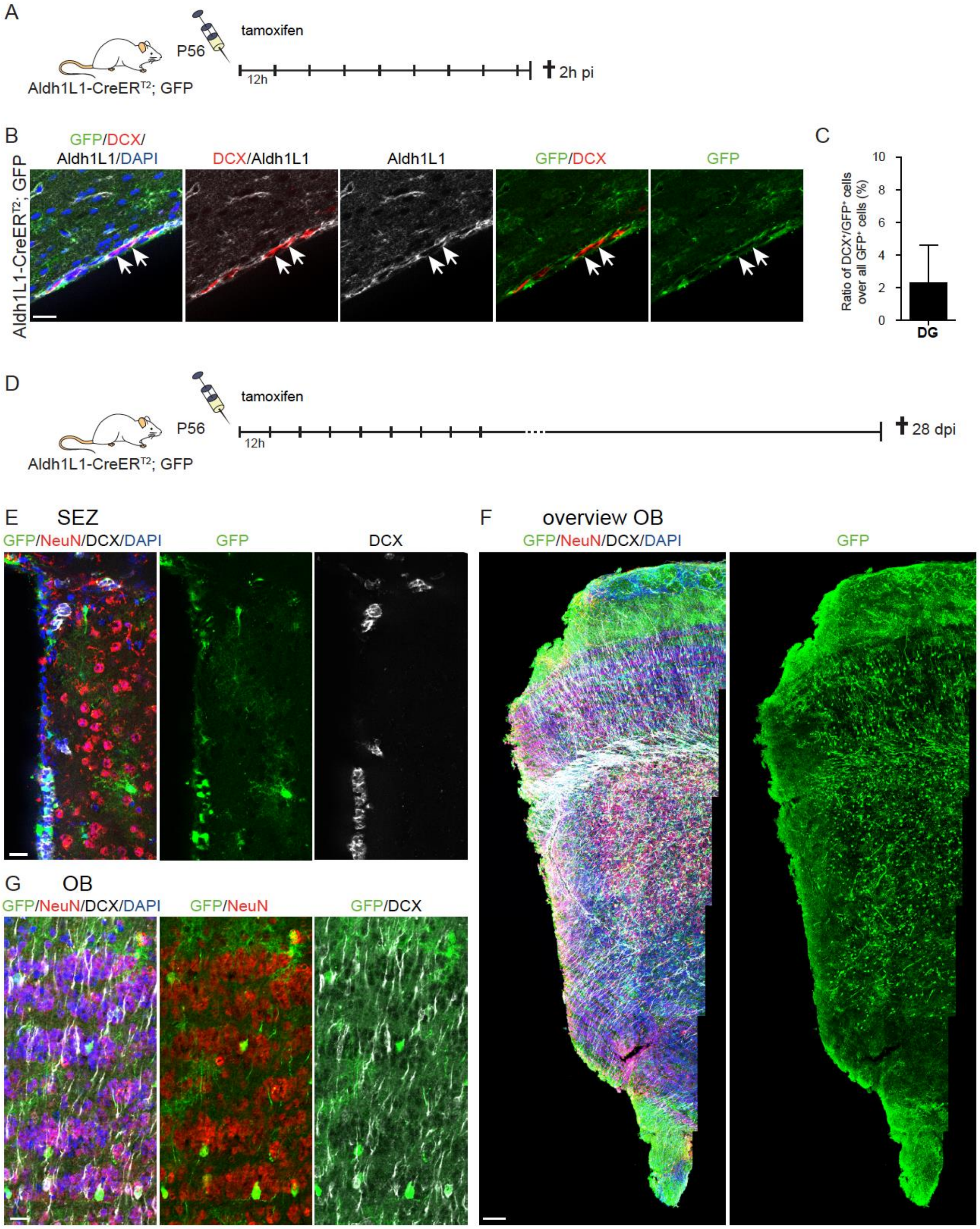

Supplemental figure 2: Recombination of Aldh1L1-CreER^{T2}; GFP mice in the SEZ, RMS and OB.

(A) Schematic drawing showing the short-term tamoxifen pulse. Here, Aldh1L1-CreER^{T2}; GFP mice were injected with tamoxifen every 12 hours (12h) for five consecutive days and killed 2 hours post-injection (2h pi). (B) Images showing the SEZ of tamoxifen-treated Aldh1L1-CreER^{T2}; GFP mice immunohistochemically stained against DCX (red) and Aldh1L1 (white). GFP is depicted in green and DAPI in blue (scale bar = 20 µm). Arrows point towards

$DCX^+/Aldh1L1^-/GFP^-$ cells in the SEZ which lie in close proximity to Aldh1L1-expressing SEZ cells. (C) Graph showing the ratio of DCX/GFP double positive NBs over all recombined (GFP^+) cells in the SEZ (mean + SEM; 2h pi). Please note that the few Cre-reporter positive NBs might not have been the initial target cell of the recombination event but rather the progeny of early recombined NSCs in the SEZ. Using the long-term tamoxifen protocol (28 dpi; D) and immunohistochemical staining with antibodies against GFP (green), DCX (white) and NeuN (red) we assessed the generation of neuronal progeny from recombined NSCs in the SEZ (E), and consequently the OB (F-G). The overview pictures (F) show part of a sagittal cut OB with part of the RMS reaching into the OB; scale bars (E, G) = 20 µm; scale bar (F) = 100 µm.
